# Assessment of early exaggerated treatment effects in orthodontic interventions using cumulative meta-analysis

**DOI:** 10.1093/ejo/cjab042

**Published:** 2021-06-29

**Authors:** Jadbinder Seehra, Daniel Stonehouse-Smith, Nikolaos Pandis

**Affiliations:** 1 Department of Orthodontics, Faculty of Dentistry, Oral & Craniofacial Sciences, King’s College London, UK; 2 Department of Orthodontics and Dentofacial Orthopedics, Dental School/Medical Faculty, University of Bern, Switzerland

## Abstract

**Background:**

The reported initial strong treatment effects reported in early trials that are refuted in subsequent future studies assessing the same interventions have been attributed to novelty bias. The aim of this study was to determine whether there is any evidence of novelty bias in the reported treatment effects of orthodontics interventions.

**Materials and methods:**

Relevant orthodontic systematic review (SRs) topics containing at least one meta-analysis on either binary or continuous outcomes with a minimum of three trials considered important areas in the field of orthodontic practice were identified. SR, meta-analysis, and primary study-level characteristics were extracted. Descriptive statistics were calculated at the SRs, meta-analysis, and at the individual study level. All SR and trial-level data were imported into the statistical software and all meta-analyses were replicated using the cumulative random-effects meta-analysis approach. Changes in the size and direction of the estimates between the first trial and the cumulative effect over time were recorded.

**Results:**

Forty-seven meta-analyses were included. The total number of primary studies included within these meta-analyses was 408 (*N* = 408). Overall, the final effect size estimate decreased in 29 (61.7%, *N* = 29/47) cumulative meta-analyses whilst it increased in the remaining 18 (38.3%, *N* = 18/47). No association between the level of risk of bias and the cumulative absolute effect size was evident (OR 1.00; 95% CI: 0.98, 1.03; *P* = 0.717) after adjusting for year of the primary study (*P* = 0.22).

**Conclusions:**

Clinicians should be wary of the results of trials reporting the effectiveness of new interventions as there is a possibility that the reported effect size will be often exaggerated.

## Introduction

The conduct of orthodontic trials has increased substantially within the literature with just under 1100 trials published during a 10-year period (1). Despite clinical trials representing high-level evidence that can inform clinical practice, they are prone to several forms of bias which can distort the direction and the size of the effect estimates (2). The effect estimates indicate in a clinically relevant manner how effective an intervention is and together with the associated confidence intervals constitute a better way to communicate clinical relevance compared to testing for statistical significance (3). Biases can be exaggerated in the results of trials assessing the effectiveness of new interventions. The initial strong treatment effects reported in early trials can be refuted in subsequent future studies assessing the same interventions (4, 5–10). The term novelty bias has been introduced to account for differences between the reporting of initially exaggerated treatment effects which are then not supported in future studies (6, 11). Novelty bias has been proposed to occur as a result of other forms of bias such as selection, outcome reporting confirmation, and ‘hot stuff’ bias having a greater impact when the intervention is new (11).

The term cumulative meta-analysis depicts a meta-analytical approach where prospectively as the results of new relevant trials become available, previous meta-analysis pooled estimates are updated (12). This analysis describes statistically how the evidence base can evolve regarding a particular intervention (13). Previous cumulative meta-analyses have reported how initially favourable estimates have either decreased, increased, or undergone no change as the evidence-based has evolved through study replications (13). Cumulative meta-analyses can also be used as a tool to identify research waste (14), reinforce the need for the design of new studies to be informed by relevant systematic reviews (SRs) (15) and the results of new studies to be reported in the context of updated SRs of similar studies (16).

The extent of novelty bias within orthodontic trials or studies is unknown. Using the cumulative meta-analysis approach, the aim of the current investigation was, as the evidence from primary studies or trials accumulates, to examine whether there is any evidence of novelty bias in the reported treatment effects of orthodontics interventions.

## Methods

### Eligibility criteria

Relevant orthodontic SR topics which were considered important areas in the field of orthodontic practice were initially identified from published literature (17, 18). Further contemporary SR topics were also identified using an electronic database search. The final selected topics were agreed by consensus between two authors (JS and NP). To be included, the SR should include at least one meta-analysis on either binary or continuous outcomes containing a minimum of three trials. Furthermore, only English language and SR reporting interventional procedures involving human participants were included. Where multiple versions of the same SR existed, the latest version was selected.

### Search of further SRs and primary studies

Both an electronic database search (Medline via PubMed) and search of Cochrane Library were undertaken using the following search terms: ‘orthodontic’ AND ‘systematic review’ OR ‘meta-analysis’ to identify further contemporary SR topics not covered in the published literature (17, 18). All titles and abstracts were initially screened by two authors (JS and NP). Full-text articles of abstracts meeting the inclusion criteria were retrieved and further assessed. For the selected SRs with a minimum of three studies, additional recent randomized clinical trials (primary studies) not included in the meta-analysis but with consistent aims and outcome measures to the SR were searched. Again, an electronic database search (Medline via PubMed) was undertaken using the following search terms and filters: ‘randomized clinical trial’ AND ‘orthodontics’ to identify further primary studies. All titles and abstracts were initially screened in duplicate (JS and NP). Any disagreements in the selection of the final SRs and additional trials were resolved by discussion among two authors (JS and NP).

### Data extraction

Study characteristics were extracted from the forest plots, tables, and text of the final included SRs. A pre-piloting process of five SRs was undertaken to ensure consistency between authors (JS, DSS, and NP) regarding data extraction, interpretation of both data variables, and forest plots independently. Once 100% agreement had been achieved, all study characteristics were then extracted by a single author (DSS) and entered into a pre-piloted Microsoft Excel^®^ (Microsoft, Redmond, Washington, USA) data collection sheet. A second author (JS) independently reviewed the collected data. Any disagreements were resolved by discussion.

At the SR level, the following information was extracted: number of authors, continent of corresponding author, year of publication, and PROSPERO registration at the meta-analysis level, the following were collected: number of primary studies included, type of effect measure (continuous or binary), and estimates with 95% CIs. The following information from the SRs at the primary study level were also extracted: year of publication and risk of bias assessment, number of patients, number of arms, type of outcome (binary or continuous), sample size, means, and standard deviation or number of events, where applicable, per treatment group, estimates, 95% CIs and *P*-values <0.05 of the contributing studies. For all newly identified trials, a risk of bias assessment was undertaken using the Cochrane risk-of-bias tool for randomized trials (19). This was conducted independently by two authors (JS and DSS). Any disagreements were resolved by discussion. When more than one meta-analysis was present, the meta-analysis directly related to the main outcome of the study was selected. When two or more meta-analyses were related to the main outcome, we selected the meta-analysis with the greatest number of primary studies included. All SR and trial-level data were imported to the statistical software and all meta-analyses were replicated using the cumulative meta-analysis approach.

### Statistical analysis

Descriptive statistics were calculated at the SR, meta-analysis, and at the individual study level. All SR and trial-level data were imported to the statistical software and all meta-analyses were replicated using the cumulative random-effects meta-analysis approach. Changes in the estimates between the first study and the cumulative pooled estimated were represented in barplots. For the binary outcomes, the estimates were log-transformed before plotting in order to improve visualization. Ordinal logistic regression was used to assess the absolute cumulative effect adjusted for year of the trial on the probability of belonging to high, unclear, or low risk of bias. All analyses were conducted using Stata 16.1 (StataCorp, College Station, Texas, USA).

## Results

**Figure 1.**
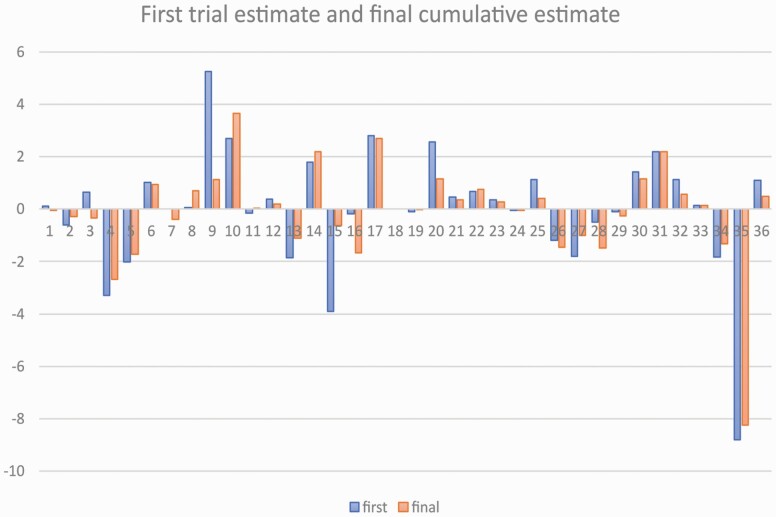
Barplot for the estimate of the first study (blue) and the cumulative estimate (orange) for meta-analyses with continuous outcomes.

**Figure 2.**
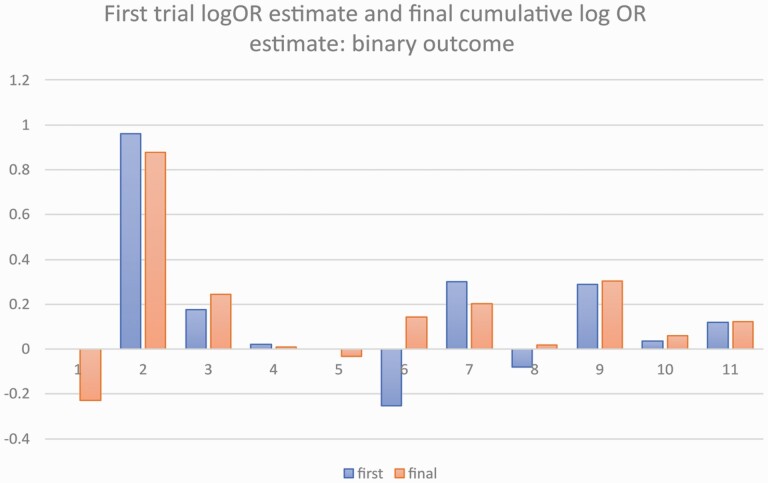
Barplot for the estimate of the first study (blue) and the cumulative estimate (orange) for meta-analyses with binary outcomes. Estimates have been log-transformed.

Forty-seven meta-analyses were included in this study (Supplementary Table 1). At the SR level, the most frequent year of publication were 2017 (19.1%) and 2018 (19.1%), with the corresponding author based in Europe (42.3%) and most SRs not being registered with PROSPERO (59.6%). The median number of SR authors was 5 (IQR 4–6). The title of each meta-analysis is provided in Supplementary Table 1. Out of the 47 meta-analyses, 36 and 11 reported continuous and binary outcomes, respectively (Table 1). Twelve additional primary studies or trials (Supplementary Table 1) were identified and hence the total number of primary studies included within the meta-analyses was 408 (*N* = 408). Overall, the final effect size estimate decreased in 29 (61.7%, *N* = 29/47) cumulative meta-analyses whilst it increased in the remaining 18 (38.3%, *N* = 18/47) (Supplementary Table 2). For continuous outcomes, in 24 cumulative meta-analyses, the final effect size estimate decreased (*N* = 24/36) (Figure 1). Conversely, in analyses reporting binary outcomes, the final effect size estimate decreased in five cumulative meta-analyses (*N* = 5/11) (Figure 2). No association between the level of risk of bias and the cumulative absolute effect size was evident (OR 1.00; 95% CI: 0.98, 1.03; *P* = 0.717) after adjusting for year of the primary study (likelihood ratio test *P*-value = 0.22).

**Table 1.** Descriptive statistics for the included systematic reviews.

Characteristic	*N* (%)
Year of publication	
2010	1 (2.1)
2012	2 (4.3)
2013	2 (4.3)
2014	7 (14.9)
2015	4 (8.5)
2016	6 (12.8)
2017	9 (19.1)
2018	9 (19.1)
2019	6 (12.8)
2020	1 (2.1)
Continent of corresponding author	
Europe	20 (42.5)
Americas	10 (21.3)
Asia or other	17 (36.2)
Registration	
Yes	19 (40.4)
No	28 (59.6)
Total	47 (100.0)

## Discussion

**Figure 3.**
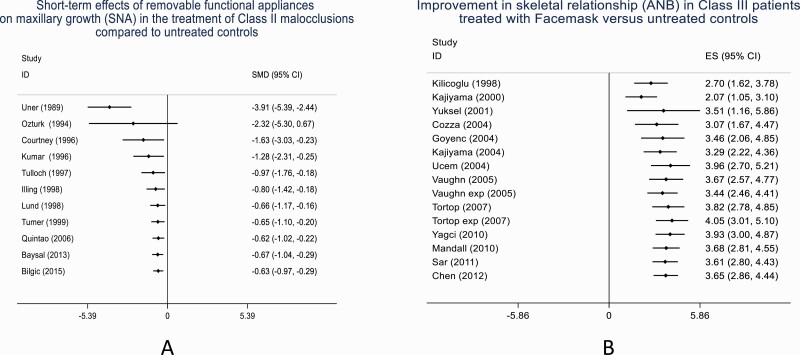
Compared to the initial effect size estimate, decrease in final absolute treatment effect size estimate (A) and increase in final absolute treatment effect size estimate (B). Although the result is still statistically significant, its clinical relevance is less evident.

The findings based on the change between the initial and the cumulative meta-analysis pooled estimates have shown evidence of exaggeration of initial treatment effects or reporting of novel agent effects (novelty bias) in orthodontic interventions. Overall, in 61.7% (*N* = 29/47) cumulative meta-analysis, the final treatment effect size estimate decreased compared to the reported first study treatment effect size estimate (Figure 3A and 3B). Similar results have been reported elsewhere. In the investigation of highly cited studies, in 16% of studies, the effects of the first trial were stronger than those of subsequent studies (20). This observation is supported by the findings of a cumulative network meta-analysis undertaken regarding the effectiveness of anti-depressants. Despite reported initial stronger effects of newly approved drugs, the effect estimate tended to decrease and stabilize over time with the addition of new evidence (21). Conversely, in 38.3% of cumulative meta-analysis, an opposite effect was observed with the final absolute treatment effect size estimate increased compared to the first reported absolute treatment effect size estimate (Figure 3B). This trend has been previously reported but tends to be fewer and weaker than the finding of the final absolute effect size decreasing (13). Within the medical literature, evidence of novelty bias has been reported (4–8). Using a multiple treatment meta-analysis model, the presence of novelty bias has been reported to result in an exaggeration of cancer treatment effect size estimate by 6% (6). Additionally in the assessment of randomized clinical trials of lipid-lowering anti-glaucoma drugs, the effectiveness of three out of four of the interventions decreased over time (5). In summary, based on the meta-analyses of clinical trials, the presence of novelty bias can result in an intervention appearing to be between 2% and 27% better when the treatment is novel (11).

Inflated newly reported effects compared to the true effect size may be the result of a combination of biases present in the study methodology including the conduct and reporting of a trial or study (22). Regarding study design, observational studies tend to report exaggerated effects which are often contradicted by the results of randomized clinical trials (23). More importantly, if replication of study design can be achieved, the results of such studies are more likely to contradict the reported initial stronger results over time (20). However, this has to be mitigated by the finding that reproducibility of research study design is both sub-optimal (24) and undertaken infrequently (25). The decrease in absolute treatment effect size estimate appears to be independent of the level of risk of bias within the primary study. It could be assumed that novelty bias exists as a result of the poor-quality studies. However, assumptions made regarding trial quality based on reported trial aspects which may deviate from what was actually done in each trial is not straightforward. Additionally, the relationship between trial quality and novelty bias has not been established (6). It should be remembered that if subsequent trials or studies contradict the findings of initial studies it does not mean that these studies were wrong in the first place and that later studies were larger or employed a controlled methodology (20). Indeed, increasing the sample size does not necessarily result in a change in the direction of the results but can increase precision. The latter explains the finding in the current study where a decreased effect from the cumulative estimate can be statistically significant whereas in the initial trial a larger effect is not significant. Alternative reasons for the overserved differences could be the differences in eligibility criteria or the use of additional interventions (26).

To reduce novelty bias effects, recommendations have been suggested (11). Investigators of new or novel interventions should be encouraged to state if the reported observed effect could be the result of novelty bias. In addition, when reporting the results of novel interventions analytical methods that correct for the anticipated inflation should be used, strict protocols for analyses can be employed and complete and transparent reporting of all results should be pre-requisite (22). Replication of studies should be encouraged (22). In this event, investigators who test the same hypotheses in future studies and report less favourable results should try to identify and explain factors that may account for the differences between the studies (11). Clinicians can also have a role in identifying potential novelty bias when interpreting the results of new interventions and should exhibit caution regarding newly discovered effect sizes (22). Furthermore, adherence to reporting guidelines by investigators should be encouraged to promote transparency in the conduct and reporting of studies (27).

Where a minimum of three studies/trials were included in the meta-analysis, an attempt was made by the authors to identity further primary studies which were consistent with the aims and outcome measures of the SR. The aim of this approach was to reduce potential uncertainty of the results by increasing the number of primary studies and range of publication dates. These studies were searched independently by two authors to reduce potential selection bias. The decision to select orthodontic SR topics which were considered important areas in the field of orthodontic practice was based on discussion between two authors (JS and NP). Although this may have led to potential selection bias, this was minimized during the selection process as both authors referred to literature documenting topics in orthodontics where meta-analyses have been commonly undertaken (17, 18). Meta-analyses published in only English were only included which can impact the generalizability of the results.

## Conclusions

To our knowledge, this is the first study in the field of orthodontics to address the topic of novelty bias using a cumulative meta-analysis approach. In approximately 60% meta-analyses, the final effect size estimate of the treatment intervention decreased as the evidence from primary studies evolved. There was no association between the level of risk of bias and the cumulative absolute effect size. Clinicians should be wary of the results of trials reporting the effectiveness of new interventions as there is a possibility that the reported effect size will be often exaggerated.

## Supplementary material

Supplementary material is available at the *European Journal of Orthodontics* online.

Supplementary Table 1 Titles of included systematic reviews and additional primary studies or trials.

Supplementary Table 2 Outcomes, comparison groups, number of studies included, period in years, risk of bias, effect measure and 95% CIs for first study and cumulative estimate, statistical significance, and change in direction of the effect size between initial and cumulative estimate for the included meta-analyses.

cjab042_suppl_Supplementary_Table_1

cjab042_suppl_Supplementary_Table_2

## Data Availability

The data underlying this article are available in the article and in its online supplementary material.
