# Supplementary material for: Assessment of early exaggerated treatment effects in orthodontic interventions using cumulative meta-analysis
Source: Eur J Orthod. 2021 Jun 29;43(5):601–5. doi: 10.1093/ejo/cjab042 (PMC8633600; doi:10.1093/ejo/cjab042)
Supplement: cjab042_suppl_Supplementary_Table_1 [file cjab042_suppl_supplementary_table_1.docx]

| **Title** | **Year of publication** |
| --- | --- |
| Bakdach, W. and Hadad, R. (2020). Effectiveness of low-level laser therapy in accelerating the orthodontic tooth movement: A systematic review and meta-analysis. Dental and medical problems, 57, 73–94. https://doi.org/10.17219/dmp/112446 | 2020 |
| Elmotaleb, M., Elnamrawy, M. M., Sharaby, F., Elbeialy, A. R., and ElDakroury, A. (2019). Effectiveness of using a Vibrating Device in Accelerating Orthodontic Tooth Movement: A Systematic Review and Meta-Analysis. Journal of International Society of Preventive & Community Dentistry, 9, 5–12.  Additional primary studies or trials   1. Miles, P., Fisher, E., and Pandis, N. (2018). Assessment of the rate of premolar extraction space closure in the maxillary arch with the AcceleDent Aura appliance vs no appliance in adolescents: A single-blind randomized clinical trial. American journal of orthodontics and dentofacial orthopedics,153, 8–14. 2. Taha, K., Conley, R. S., Arany, P., Warunek, S., and Al-Jewair, T. (2020). Effects of mechanical vibrations on maxillary canine retraction and perceived pain: a pilot, single-center, randomized-controlled clinical trial. Odontology, 108, 321–330. 3. Katchooi, M., Cohanim, B., Tai, S., Bayirli, B., Spiekerman, C., and Huang, G. (2018). Effect of supplemental vibration on orthodontic treatment with aligners: A randomized trial. American journal of orthodontics and dentofacial orthopedics, 153, 336–346. 4. Pavlin, D., Anthony, R., Raj, V., and Gakunga P.T. (2015). Cyclic loading (vibration) accelerates tooth movement in orthodontic patients: a double-blind, randomized controlled trial. Semin Orthod, 21, 87-94. | 2019 |
| Alhadainy, H. A., Flores-Mir, C., Abdel-Karim, A. H., Crossman, J., and El-Bialy, T. (2019). Orthodontic-induced External Root Resorption of Endodontically Treated Teeth: A Meta-analysis. Journal of endodontics, 45, 483–489.  Additional primary studies or trials   1. Kreia, T.B., Tanaka, O., Lara, F., Camargo, E.S., Maruo, H., and Westphalen VPD (2005) Avaliação da reabsorção radicular após a movimenta- ção ortodôntica em dentes tratados endodonticamente/Evaluation of root resorption after orthodontic treatment in endodontically treated teeth. Rev Odonto Ciênc ,20,50–56 | 2019 |
| Dab, S., Chen, K., and Flores-Mir, C. (2019). Short- and long-term potential effects of accelerated osteogenic orthodontic treatment: A systematic review and meta-analysis. Orthodontics & craniofacial research, 22, 61–68. | 2019 |
| Alharbi, F., Almuzian, M., and Bearn, D. (2019). Anchorage effectiveness of orthodontic miniscrews compared to headgear and transpalatal arches: a systematic review and meta-analysis. Acta odontologica Scandinavica, 77, 88–98. | 2019 |
| Tasios, T., Papageorgiou, S. N., Papadopoulos, M. A., Tsapas, A., and Haidich, A. B. (2019). Prevention of orthodontic enamel demineralization: A systematic review with meta-analyses. Orthodontics & craniofacial research, 22, 225–235. | 2019 |
| Becker, K., Pliska, A., Busch, C., Wilmes, B., Wolf, M., and Drescher, D. (2018). Efficacy of orthodontic mini implants for en masse retraction in the maxilla: a systematic review and meta-analysis. International journal of implant dentistry, 4, 35. | 2018 |
| Batista, K. B., Thiruvenkatachari, B., Harrison, J. E., and O'Brien, K. D. (2018). Orthodontic treatment for prominent upper front teeth (Class II malocclusion) in children and adolescents. The Cochrane database of systematic reviews, 3, CD003452. | 2018 |
| Almuzian, M., McConnell, E., Darendeliler, M. A., Alharbi, F., and Mohammed, H. (2018). The effectiveness of alternating rapid maxillary expansion and constriction combined with maxillary protraction in the treatment of patients with a class III malocclusion: a systematic review and meta-analysis. Journal of orthodontics, 45, 250–259. | 2018 |
| Mohammed, H., Wafaie, K., Rizk, M. Z., Almuzian, M., Sosly, R., and Bearn, D. R. (2018). Role of anatomical sites and correlated risk factors on the survival of orthodontic miniscrew implants: a systematic review and meta-analysis. Progress in orthodontics, 19, 36. | 2018 |
| Mohammed, H., Rizk, M. Z., Wafaie, K., Ulhaq, A., and Almuzian, M. (2019). Reminders improve oral hygiene and adherence to appointments in orthodontic patients: a systematic review and meta-analysis. European journal of orthodontics, 41, 204–213. | 2018 |
| Grisar, K., Chaabouni, D., Romero, L., Vandendriessche, T., Politis, C., and Jacobs, R. (2018). Autogenous transalveolar transplantation of maxillary canines: a systematic review and meta-analysis. European journal of orthodontics, 40, 608–616. | 2018 |
| Wei, H., Liu, Z., Zang, J., and Wang, X. (2018). Surgery-first/early-orthognathic approach may yield poorer postoperative stability than conventional orthodontics-first approach: a systematic review and meta-analysis. Oral surgery, oral medicine, oral pathology and oral radiology, 126, 107–116. | 2018 |
| Alyammahi, A. S., Kaklamanos, E. G., and Athanasiou, A. E. (2018). Effectiveness of extraction of primary canines for interceptive management of palatally displaced permanent canines: a systematic review and meta-analysis. European journal of orthodontics, 40, 149-156. | 2018 |
| Schwartz, M., Acosta, L., Hung, Y. L., Padilla, M., and Enciso, R. (2018). Effects of CPAP and mandibular advancement device treatment in obstructive sleep apnea patients: a systematic review and meta-analysis. Sleep & breathing = Schlaf & Atmung, 22, 555–568. | 2018 |
| Xiang, M., Hu, B., Liu, Y., Sun, J., and Song, J. (2017). Changes in airway dimensions following functional appliances in growing patients with skeletal class II malocclusion: A systematic review and meta-analysis. International journal of pediatric otorhinolaryngology, 97, 170–180. | 2017 |
| Rongo, R., D'Antò, V., Bucci, R., Polito, I., Martina, R., and Michelotti, A. (2017). Skeletal and dental effects of Class III orthopaedic treatment: a systematic review and meta-analysis. Journal of oral rehabilitation, 44, 545–562. | 2017 |
| Janson, G., Aliaga-Del Castillo, A., and Niederberger, A. (2017). Changes in apical base sagittal relationship in Class II malocclusion treatment with and without premolar extractions: A systematic review and meta-analysis. The Angle orthodontist, 87, 338–355. | 2017 |
| Diar-Bakirly, S., Feres, M. F., Saltaji, H., Flores-Mir, C., and El-Bialy, T. (2017). Effectiveness of the transpalatal arch in controlling orthodontic anchorage in maxillary premolar extraction cases: A systematic review and meta-analysis. The Angle orthodontist, 87, 147–158. | 2017 |
| Yang, X., Su, N., Shi, Z., Xiang, Z., He, Y., Han, X., and Bai, D. (2017). Effects of self-ligating brackets on oral hygiene and discomfort: a systematic review and meta-analysis of randomized controlled clinical trials. International journal of dental hygiene, 15, 16–22. | 2017 |
| Feres, M. F., Abreu, L. G., Insabralde, N. M., de Almeida, M. R., and Flores-Mir, C. (2017). Effectiveness of open bite correction when managing deleterious oral habits in growing children and adolescents: a systematic review and meta-analysis. European journal of orthodontics, 39, 31–42. | 2017 |
| Monk, A. B., Harrison, J. E., Worthington, H. V., and Teague, A. (2017). Pharmacological interventions for pain relief during orthodontic treatment. The Cochrane database of systematic reviews, 11, CD003976. | 2017 |
| Yi, J., Ge, M., Li, M., Li, C., Li, Y., Li, X., and Zhao, Z. (2017). Comparison of the success rate between self-drilling and self-tapping miniscrews: a systematic review and meta-analysis. European journal of orthodontics, 39, 287–293. | 2017 |
| Guo, R., Lin, Y., Zheng, Y., and Li, W. (2017). The microbial changes in subgingival plaques of orthodontic patients: a systematic review and meta-analysis of clinical trials. BMC oral health, 17, 90. | 2017 |
| Neelapu, B. C., Kharbanda, O. P., Sardana, H. K., Balachandran, R., Sardana, V., Kapoor, P., Gupta, A., and Vasamsetti, S. (2017). Craniofacial and upper airway morphology in adult obstructive sleep apnea patients: A systematic review and meta-analysis of cephalometric studies. Sleep medicine reviews, 31, 79–90. | 2016 |
| Cheng, H. C., Wang, Y. C., Tam, K. W., and Yen, M. F. (2016). Effects of tooth extraction on smile esthetics and the buccal corridor: A meta-analysis. Journal of dental sciences, 11, 387–393. | 2016 |
| Yi, J., Li, M., Li, Y., Li, X., and Zhao, Z. (2016). Root resorption during orthodontic treatment with self-ligating or conventional brackets: a systematic review and meta-analysis. BMC oral health, 16, 125. | 2016 |
| Nucera, R., Lo Giudice, A., Rustico, L., Matarese, G., Papadopoulos, M. A., and Cordasco, G. (2016). Effectiveness of orthodontic treatment with functional appliances on maxillary growth in the short term: A systematic review and meta-analysis. American journal of orthodontics and dentofacial orthopedics, 149, 600–611.e3. | 2016 |
| Elkordy, S. A., Aboelnaga, A. A., Fayed, M. M., AboulFotouh, M. H., and Abouelezz, A. M. (2016). Can the use of skeletal anchors in conjunction with fixed functional appliances promote skeletal changes? A systematic review and meta-analysis. European journal of orthodontics, 38, 532–545. | 2016 |
| Zymperdikas, V. F., Koretsi, V., Papageorgiou, S. N., and Papadopoulos, M. A. (2016). Treatment effects of fixed functional appliances in patients with Class II malocclusion: a systematic review and meta-analysis. European journal of orthodontics, 38, 113–126.  Additional primary studies or trials   1. Eissa, O., El-Shennawy, M., Gaballah, S., El-Meehy, G., and El Bialy, T. (2017). Treatment outcomes of Class II malocclusion cases treated with miniscrew-anchored Forsus Fatigue Resistant Device: A randomized controlled trial. The Angle orthodontist, 87, 824–833. 2. Elkordy, S. A., Abouelezz, A. M., Fayed, M., Aboulfotouh, M. H., and Mostafa, Y. A. (2019). Evaluation of the miniplate-anchored Forsus Fatigue Resistant Device in skeletal Class II growing subjects: A randomized controlled trial. The Angle orthodontist, 89, 391–403. | 2016 |
| Zhou, Q., Ul Haq, A. A., Tian, L., Chen, X., Huang, K., and Zhou, Y. (2015). Canine retraction and anchorage loss self-ligating versus conventional brackets: a systematic review and meta-analysis. BMC oral health, 15, 136. | 2015 |
| Perinetti, G., Primožič, J., Franchi, L., and Contardo, L. (2015). Treatment Effects of Removable Functional Appliances in Pre-Pubertal and Pubertal Class II Patients: A Systematic Review and Meta-Analysis of Controlled Studies. PloS one, 10, e0141198. | 2015 |
| Perinetti, G., Primožič, J., Furlani, G., Franchi, L., and Contardo, L. (2015). Treatment effects of fixed functional appliances alone or in combination with multibracket appliances: A systematic review and meta-analysis. The Angle orthodontist, 85, 480–492. | 2015 |
| Fleming, P. S., Fedorowicz, Z., Johal, A., El-Angbawi, A., and Pandis, N. (2015). Surgical adjunctive procedures for accelerating orthodontic treatment. The Cochrane database of systematic reviews, 2015, CD010572.  Additional primary studies or trials   1. Babanouri, N., Ajami, S., and Salehi, P. (2020). Effect of mini-screw-facilitated micro-osteoperforation on the rate of orthodontic tooth movement: a single-center, split-mouth, randomized, controlled trial. Progress in orthodontics, 21, 7. 2. Fattori, L., Sendyk, M., de Paiva, J. B., Normando, D., and Neto, J. R. (2020). Micro-osteoperforation effectiveness on tooth movement rate and impact on oral health related quality of life. The Angle orthodontist, 90, 640–647. 3. Gulduren, K., Tumer, H., and Oz, U. (2020). Effects of micro-osteoperforations on intraoral miniscrew anchored maxillary molar distalization : A randomized clinical trial. Effekte von Mikroosteoperforationen auf die minischraubenunterstützte molare Distalisierung im Oberkiefer : Eine randomisierte klinische Studie. Journal of orofacial orthopedics = Fortschritte der Kieferorthopadie : Organ/official journal Deutsche Gesellschaft fur Kieferorthopadie, 81, 126–141. 4. Sivarajan, S., Doss, J. G., Papageorgiou, S. N., Cobourne, M. T., and Wey, M. C. (2019). Mini-implant supported canine retraction with micro-osteoperforation: A split-mouth randomized clinical trial. The Angle orthodontist, 89, 183–189. | 2015 |
| Yang, X., Li, C., Bai, D., Su, N., Chen, T., Xu, Y and Han X. (2014). Treatment effectiveness of Fränkel function regulator on the Class III malocclusion: a systematic review and meta-analysis. Am J Orthod Dentofacial Orthop, 146,143-54. | 2014 |
| Papageorgiou, S. N., Konstantinidis, I., Papadopoulou, K., Jäger, A., and Bourauel, C. (2014). Clinical effects of pre-adjusted edgewise orthodontic brackets: a systematic review and meta-analysis. European journal of orthodontics, 36, 350–363. | 2014 |
| Pandis, N., Fleming, P. S., Spineli, L. M., and Salanti, G. (2014). Initial orthodontic alignment effectiveness with self-ligating and conventional appliances: a network meta-analysis in practice. American journal of orthodontics and dentofacial orthopedics, 145(4 Suppl), S152–S163. | 2014 |
| Papageorgiou, S. N., Kutschera, E., Memmert, S., Gölz, L., Jäger, A., Bourauel, C., and Eliades, T. (2017). Effectiveness of early orthopaedic treatment with headgear: a systematic review and meta-analysis. European journal of orthodontics, 39, 176–187. | 2014 |
| Dalessandri, D., Salgarello, S., Dalessandri, M., Lazzaroni, E., Piancino, M., Paganelli, C., Maiorana, C., and Santoro, F. (2014). Determinants for success rates of temporary anchorage devices in orthodontics: a meta-analysis (n > 50). European journal of orthodontics, 36, 303–313. | 2014 |
| Jambi, S., Walsh, T., Sandler, J., Benson, P. E., Skeggs, R. M., and O'Brien, K. D. (2014). Reinforcement of anchorage during orthodontic brace treatment with implants or other surgical methods. The Cochrane database of systematic reviews, 2014, CD005098. | 2014 |
| Zhou, Y., Long, H., Ye, N., Xue, J., Yang, X., Liao, L., and Lai, W. (2014). The effectiveness of non-surgical maxillary expansion: a meta-analysis. European journal of orthodontics, 36, 233–242. | 2014 |
| Fleming, P. S., Eliades, T., Katsaros, C., and Pandis, N. (2013). Curing lights for orthodontic bonding: a systematic review and meta-analysis. American journal of orthodontics and dentofacial orthopedics, 143(4 Suppl), S92–S103. | 2013 |
| Jambi, S., Thiruvenkatachari, B., O'Brien, K. D., and Walsh, T. (2013). Orthodontic treatment for distalising upper first molars in children and adolescents. The Cochrane database of systematic reviews, 2013, CD008375. | 2013 |
| Fleming, P. S., Johal, A., and Pandis, N. (2012). Self-etch primers and conventional acid-etch technique for orthodontic bonding: a systematic review and meta-analysis. American journal of orthodontics and dentofacial orthopedics, 142, 83–94.  Additional primary studies or trials   1. Shah, J., and Chadwick, S. (2009). Comparaison entre un système de collage orthodontique en une étape et un système de collage en deux étapes : revue de littérature et résultats d'un essai clinique randomisé [Comparison of 1-stage orthodontic bonding systems and 2-stage bonding systems: a review of the literature and the results of a randomized clinical trial]. L' Orthodontie francaise, 80, 167–178. | 2012 |
| Vilani, G. N., Mattos, C. T., de Oliveira Ruellas, A. C., and Maia, L. C. (2012). Long-term dental and skeletal changes in patients submitted to surgically assisted rapid maxillary expansion: a meta-analysis. Oral surgery, oral medicine, oral pathology and oral radiology, 114, 689–697. | 2012 |
| Fricton, J., Look, J. O., Wright, E., Alencar, F. G., Jr, Chen, H., Lang, M., Ouyang, W., and Velly, A. M. (2010). Systematic review and meta-analysis of randomized controlled trials evaluating intraoral orthopedic appliances for temporomandibular disorders. Journal of orofacial pain, 24, 237–254. | 2010 |

**Supplemental Table 1** Titles of included systematic reviews
