# Supplementary material for: Assessment of early exaggerated treatment effects in orthodontic interventions using cumulative meta-analysis
Source: Eur J Orthod. 2021 Jun 29;43(5):601–5. doi: 10.1093/ejo/cjab042 (PMC8633600; doi:10.1093/ejo/cjab042)
Supplement: cjab042_suppl_Supplementary_Table_2 [file cjab042_suppl_supplementary_table_2.docx]

| **Outcome and comparison group of each meta-analysis** | **Number of studies included (N)/ Period (Years)** | **Risk of Bias (N)** | **Effect measure** | **First study estimate (95% CI)** | **Cumulative estimate**  **(95% CI)** | **Change** |
| --- | --- | --- | --- | --- | --- | --- |
| Effectiveness of vibrational devices in increasing the rate of orthodontic tooth movement (mm/per month) compared to non-vibrational control group | 7 (2012-2020) | High (5)  Low (2) | SMD | 0.08  (-0.41,0.57)  NS | -0.01  (-0.26,0.25)  NS | Decrease |
| Risk of root resorption of endontically treated teeth compared to vital teeth following orthodontic treatment | 8 (1990-2016) | High (8) | SMD | -0.59  (-1.02, -0.16)  sig | -0.28  (-0.57, 0.02)  NS | Decrease |
| Effect on periodontal condition (probing depths) in patients undergoing accelerated osteogenic orthodontic treatment | 4 (2012-2017) | High (2)  Unclear (2) | SMD | 0.65  (-0.25,1.55)  NS | -0.34  (-1.68, 1.01)  NS | Decrease |
| Antero-posterior (AP) anchorage loss during en masse retraction in the maxilla (mini-implant versus conventional methods) | 7 (2008-2014) | High (4)  Low (1)  Unclear (3) | SMD | -3.30  (-4.32, -2.28)  Sig | -2.69  (-3.58, -1.81)  Sig | Decrease |
| Anchorage effectiveness (mesiodistal movement of upper first molar) of orthodontic miniscrews compared to conventional methods (headgear and transpalatal arches) | 8 (2008-2014) | High (3)  Low (4)  Unclear (1) | SMD | -2.01  (-2.82, -1.20)  Sig | -1.73  (-2.87, -0.59)  Sig | Decrease |
| Effect on maxilla (SNA) in growing Class III patients undergoing Protraction Facial Mask with either alternate Rapid Maxillary Expansion and Constriction or conventional Rapid Maxillary Expansion | 4 (2012-2017) | Low (1)  Unclear (3) | SMD | 1.03  (0.09, 1.97)  Sig | 0.94  (0.55, 1.32)  Sig | Decrease |
| Failure rates of miniscrews related to their specific insertion site (left versus right side) | 15 (2010-2017) | High (1)  Low (4)  Unclear (10) | OR | 1.00  (0.06, 16.89)  NS | 0.59  (0.36, 0.97)  Sig | Increase |
| Effectiveness of reminders on oral hygiene (Short term plaque scores) | 5 (2014-2018) | Low (3)  Unclear (2) | SMD | 0.00  (-0.60, 0.60)  NS | -0.38  (-0.67, -0.10)  Sig | Increase |
| Survival rate (2-5 years) of permanent maxillary canines following transalveolar transplantation | 8 (1983-2010) | Unclear (8) | OR | 9.12  (5.23, 15.88)  Sig | 7.53  (3.14, 18.06)  Sig | Decrease |
| Stability (postoperative horizontal changes of pogonion) of surgery first compared to conventional orthodontics first treatment in adult patients | 11 (2010-2017) | High (11) | SMD | 0.07  (-0.63, 0.77)  NS | 0.70  (0.30, 1.10)  Sig | Increase |
| Effectiveness of interceptive extraction of primary canines versus no extraction to normalise the eruption of palatally displaced maxillary permanent canines | 5 (2004-2015) | High (3)  Low (2) | RR | 1.50  (-0.16, 3.16)  NS | 1.75  (1.27, 2.24)  Sig | Increase |
| Effects of functional appliances on upper airway dimensions (middle pharyngeal space) in growing Class II patients with mandibular retrognathism compared to untreated controls | 7(1998-2015) | Low (7) | SMD | 5.26  (3.93, 6.59)  Sig | 1.12  (0.30, 1.93)  Sig | Decrease |
| Improvement in skeletal relationship (ANB) in Class III patients treated with Facemask verses untreated controls | 17 (1998-2012) | High (2)  Low (1)  Unclear (14) | SMD | 2.70  (1.62, 3.78)  Sig | 3.65  (2.86, 4.44)  Sig | Increase |
| Effect on aesthetic score in patients undergoing extraction versus non-extraction orthodontic treatment | 4 (1995-2014) | Not reported (4) | SMD | -0.15  (-0.65, 0.36)  NS | 0.02  (-0.32, 0.35)  NS | Decrease |
| Root resorption of mandibular central incisors associated with conventional and self-ligation labial fixed appliances | 5 (1995-2015) | Low (1)  Unclear (4) | SMD | 0.37  (-0.13, 0.87)  NS | 0.20  (-0.05, 0.46)  NS | Decrease |
| Effect on skeletal profile (ANB) in Class II patients undergoing non-extraction orthodontic treatment compared to untreated controls | 13 (2001-2015) | Unclear (13) | SMD | -1.85  (-2.40, -1.30)  Sig | -1.12  (-1.45, -0.80)  Sig | Decrease |
| Anchorage effectiveness of the transpalatal arch (TPA) in preventing maxillary molar mesialization during retraction of the anterior teeth compared to conventional methods | 8 (2008-2014) | High (5)  Unclear (3) | SMD | 1.79  (1.16, 2.43)  Sig | 2.20  (1.46, 2.94)  Sig | Increase |
| Success rates of self-drilling versus self-tapping miniscrews when used as anchorage reinforcement devices | 6 (2009-2015) | Unclear (6) | OR | 1.05  (0.74, 1.50)  Sig | 1.02  (0.86, 1.22)  Sig | Decrease |
| Short-term effects of removable functional appliances on maxillary growth (SNA) in the treatment of Class II malocclusions compared to untreated controls | 11 (1989-2015) | High (4)  Low (3)  Unclear (4) | SMD | -3.91  (-5.39, -2.44)  Sig | -0.63  (-0.97, -0.29)  Sig | Decrease |
| Comparison of level of oral hygiene (Plaque Index) in orthodontic patients treated with either self-ligation or conventional brackets | 4 (2014) | Unclear (4) | SMD | -0.18  (-0.87, 0.50)  NS | -1.68  (-3.92, 0.56)  NS | Increase |
| Improvement in overbite in growing patients with anterior open bite following Crib therapy | 4 (1997-2013) | High (2)  Unclear (2) | SMD | 2.81  (1.66, 3.97)  Sig | 2.69  (1.49, 3.90)  Sig | Decrease |
| Dento-alveolar effects (lower incisor) in patients who undergo fixed functional appliance therapy with and without skeletal anchorage | 5 (2009-2015) | High (3)  Unclear (2) | SMD | -22.86  (-30.60, -15.12)  Sig | -1.53  (-2.76, -0.30)  Sig | Decrease |
| Comparison of rate of canine retraction in patients undergoing treatment with either self-ligating or conventional orthodontic brackets | 4 (2010-2014) | High (3)  Low (1) | SMD | -0.11  (-0.53, 0.31)  NS | -0.03  (-0.31, 0.24)  NS | Decrease |
| Total mandibular length of pubertal patients with a Class II malocclusions who undergo removable functional appliances therapy compared to untreated controls | 6 (2000-2013) | Low (1)  Unclear (5) | SMD | 2.56  (1.56, 3.55)  Sig | 1.16  (0.76, 1.56)  Sig | Decrease |
| Improvement of mandibular position (SNB) in treatment of Class II malocclusions with fixed functional appliances compared to untreated controls | 14 (2005-2019) | High (4)  Unclear (10) | SMD | 0.46  (-0.05, 0.97)  NS | 0.36  (0.11, 0.60)  Sig | Decrease |
| Total mandibular length of pubertal patients with a Class II malocclusions who undergo fixed functional appliances therapy compared to untreated controls | 6 (1999-2012) | Low (1)  Unclear (5) | SMD | 0.68  (0.23, 1.14)  Sig | 0.76  (0.48, 1.04)  Sig | Increase |
| Short-term improvement in maxillary position (SNA) in Class III patients treated with FR-3 appliance verses untreated controls | 5 (1994-2008) | Low (5) | SMD | 0.35  (-0.23, 0.92)  NS | 0.28  (-0.26, 0.82)  NS | Decrease |
| Improvement in tooth irregularity in orthodontic patients undergoing treatment with either self-ligation or conventional brackets | 6 (2005-2012) | Unclear (6) | SMD | -0.04  (-0.52, 0.45)  NS | -0.05  (-0.26, 0.16)  NS | Increase |
| Risk of orthodontic bracket failures with Halogen lights compared to either plasma arc light and light-emitting diodes (LED) | 8 (2004-2008) | Low (3)  Unclear (5) | OR | 1.00  (0.44, 2.26)  NS | 0.93  (0.74, 1.17)  NS | Increase |
| Failure of orthodontic brackets at a minimum of 6 months follow-up when bonded with either self-etching primer or two-stage etch and primer | 9 (2004-2009) | High (5)  Low (2)  Unclear (2) | ES | 0.56  (-0.10, 1.22)  NS | 1.39  (0.85, 1.94)  NS | Decrease |
| Frequency of periodontal pathogens following 3 months after orthodontic appliance placement | 4 (2008-2016) | Unclear (4) | OR | 2.00  (0.67, 5.98)  NS | 1.60  (0.77, 3.33)  NS | Decrease |
| Effectiveness of surgical adjunctive procedures in accelerating orthodontic tooth movement over 1 month (mm) compared to non- surgical adjunctive procedures | 11 (2011-2020) | High (2)  Low (5)  Unclear (4) | SMD | 1.14  (-0.14, 2.42)  NS | 0.42  (0.11, 0.74)  Sig | Decrease |
| Distal movement of upper first molars in orthodontic patients (Headgear versus Intra-oral fixed Class II correctors) | 4 (2005-2011) | Low (1)  Unclear (3) | SMD | -1.20  (-1.87, -0.53)  Sig | -1.45  (-2.74, -0.15)  Sig | Increase |
| Management of orthodontic pain at 24 hours with analgesics (Paracetamol and NSAID) vs control | 26 (2005-2016) | High (7)  Low (2)  Unclear (17) | SMD | -1.80  (-3.00, -0.60)  Sig | -1.01  (-1.38, -0.63)  Sig | Decrease |
| Orthodontic anchorage effectiveness (mesiodistal movement of the upper first molar) of conventional versus surgical methods | 7 (2007-2012) | High (1)  Low (3)  Unclear (3) | SMD | -0.49  (-1.08, 0.09)  NS | -1.49  (-2.26, -0.73)  Sig | Increase |
| Improvement in skeletal relationship (ANB) in Class II patients treated with Twin Block appliance verses other functional appliances | 6 (1998-2015) | High (3)  Low (2)  Unclear (1) | SMD | -0.09  (-0.77, 0.58)  NS | -0.27  (-0.49, -0.05)  Sig | Increase |
| Stability of inter-canine width (mm) in patients who undergo surgically assisted rapid maxillary expansion | 5 (1998-2009) | Unclear (5) | SMD | 1.42  (0.83, 2.01)  Sig | 1.15  (0.87, 1.43)  Sig | Decrease |
| Maxillary intermolar width (mm) of patients who undergo rapid maxillary expansion versus control | 6 (2000-2008) | Not reported (6) | SMD | 2.20  (1.70, 2.70)  Sig | 2.18  (1.76, 2.61)  Sig | Decrease |
| Sella - hyoid bone length (mm) in obstructive sleep apnoea patients compared to control subjects | 4 (1998-2013) | Not reported (4) | SMD | 1.12  (0.41, 1.83)  Sig | 0.57  (0.26, 0.88)  Sig | Decrease |
| Efficacy of hard stabilization appliances compared to palatal non-occluding appliances in patients with Temporomandibular Disorders | 7 (1987-2006) | Unclear (7) | logOR | 1.95  (-4.91, 8.81)  NS | 2.01  (0.81, 3.21)  Sig | Increase |
| Efficiency of initial orthodontic alignment comparing self-ligating versus conventional brackets | 6 (2006-2012) | High (3)  Low (2)  Unclear (1) | SMD | 0.13  (-0.39, 0.64)  NS | 0.14  (-0.18, 0.47)  NS | Increase |
| Effect of headgear on the maxilla (SNA) in the treatment of Class II malocclusions compared to untreated controls | 13 (1991-2010) | High (12)  Low (1) | SMD | -1.83  (-2.56, -1.10)  Sig | -1.72  (-2.23, -1.21)  Sig | Decrease |
| Prevention of orthodontic enamel decalcification (Fluoride adhesives versus conventional adhesives) | 5 (1992-2017) | High (3)  Unclear (2) | OR | 1.09  (0.45, 2.66)  NS | 1.15  (0.91, 1.46)  NS | Increase |
| Prevention of orthodontic enamel decalcification (Sealant versus no sealant) | 5 (1994-2013) | High (4)  Unclear (1) | OR | 1.32  (1.02, 1.71)  Sig | 1.33  (1.01, 1.75)  Sig | Increase |
| Effectiveness of mandibular advancement devices versus CPAP in adult obstructive sleep apnea patients (Improvement of Apnea-hypopnea Index (AHI)) | 12 (1996-2014) | High (5)  Unclear (7) | SMD | -8.79  (-14.50, -3.08)  Sig | -8.24  (-13.13, -3.35)  Sig | Decrease |
| Effectiveness of low-level laser in accelerating orthodontic upper canine retraction (mm) versus control groups over 2 months | 12 (2004-2018) | High (1)  Low (3)  Unclear (8) | SMD | 1.09  (0.88, 1.30)  Sig | 0.50  (0.29, 0.72)  Sig | Increase |
| Influence of patient’s gender (male versus females) on temporary anchorage devices failure rate | 14 (2003-2011) | Unclear (14) | OR | 0.83  (0.08, 8.43)  NS | 1.04  (0.82, 1.32)  NS | Decrease |

**Supplemental Table 2** Outcomes, comparison groups, number of studies included, period in years, risk of bias, effect measure and 95% CIs for first study and cumulative estimate, statistical significance and change in direction of the effect size between initial and cumulative estimate for the included meta-analyses.
